# Effects of Advance Care Planning on the Mental Health of Bereaved Families: A Systematic Review

**DOI:** 10.7759/cureus.82403

**Published:** 2025-04-16

**Authors:** Michihiro Tsubaki, Hideaki Aoyagi, Yoshiyasu Ito, Masahiro Kobayashi, Ai Ushiwata

**Affiliations:** 1 School of Nursing, Kitasato University, Sagamihara, JPN; 2 Faculty of Nursing, Tsuruga Nursing University, Tsuruga, JPN; 3 Faculty of Nursing, Musashino University, Tokyo, JPN; 4 School of Pharmacy, Kitasato University, Tokyo, JPN

**Keywords:** advance care planning (acp), bereaved families, end-of-life care, mental health, surrogate decision-makers, systematic review

## Abstract

Decisions regarding end-of-life care are often made by surrogate decision makers, such as family members. These decisions can place a significant psychological burden on surrogates, especially when the patients’ wishes are unclear. Advance care planning (ACP) is a patient-centered preconsultation process that is expected to improve the mental health of surrogate decision-makers. This study aimed to clarify the impact of ACP intervention on the mental health of bereaved families and verify its effectiveness. This review followed the Preferred Reporting Items for Systematic Reviews and Meta-Analyses guidelines. MEDLINE, EMBASE, PsycINFO, and Cochrane Central Register of Controlled Trials were searched for studies evaluating ACP interventions published up to 2024. Studies were independently selected by two researchers using the Covidence systematic review software (Veritas Health Innovation, Melbourne, Australia). The inclusion criteria were (i) studies evaluating the effectiveness of ACP interventions, (ⅱ) studies reporting bereaved mental health outcomes, and (ⅲ) randomized controlled trials (RCTs). Among the 2,025 studies initially identified, 12 RCTs were included. All interventions involved health professionals trained to facilitate ACP discussions before the patient died. Mental health was assessed using depression, anxiety, and posttraumatic stress disorder scales. The items for which significant differences were reported varied among the studies, but several studies reported effectiveness in treating depression in bereaved families. Furthermore, when baseline-adjusted results were included, ACP interventions reduced post-bereavement anxiety and stress experienced by families. In conclusion, depending on the targets and intervention methods, pre-death discussions may improve the mental health of bereaved families. Thus, ACP should be implemented to help bereaved families.

## Introduction and background

Decisions regarding end-of-life care are often made by surrogate decision makers, such as family members, because patients are unconscious [1]. These decisions can place a significant mental burden on surrogates, especially when the patients’ wishes are unclear [2,3]. In recent years, there has been an increase in the emphasis on end-of-life care, including care for families of patients in intensive care units (ICUs) where many patients die, and the importance of dialogue in addition to complex care has been highlighted [4,5]. Thus, communication with families in ICUs has been established as a new form of care [6]. However, many bereaved families still experience mental health problems after bereavement, and they receive insufficient care [7]. Critically ill patients in the ICU cannot be included in the dialogue; thus, to reduce the burden of decision-making on the family, it is important to have a discussion before the patient enters the ICU.

Given this background, a discussion process including advance care planning (ACP) is important for decision-making in end-of-life care, such as in ICUs. ACP helps adults of all ages and health conditions understand and share their personal values, life goals, and preferences regarding future healthcare [8]. This intervention is expected to improve the mental health of surrogates. This study aimed to clarify the impact of ACP intervention on the mental health of bereaved families and verify its effectiveness.

## Review

Material and methods

Study Design and Search Strategy

This review followed the Preferred Reporting Items for Systematic Reviews and Meta-Analyses (PRISMA) guidelines [9]. The protocol was registered in PROSPERO (CRD42022381929). MEDLINE, EMBASE, PsycINFO, and the Cochrane Central Register of Controlled Trials were searched for studies evaluating ACP interventions published until 2024. The inclusion criteria were (i) studies evaluating the effectiveness of ACP interventions, (ⅱ) studies reporting bereaved mental health outcomes, (ⅲ) and randomized controlled trials (RCTs). Studies were excluded if they were non-English publications or if the intervention target was individuals aged ≤18 years. The search strategy is summarized in Table 1.

**Table 1:** Search strategy

Search database	Search query	Result
MEDLINE (EBSCO)	("Advance Care Planning"[Mesh] OR "Decision Making"[Mesh] OR "Advance Directives"[Mesh] OR "Living Wills"[Mesh]) AND ("Family Relations"[Mesh] OR "Family Health"[Mesh] OR "Family"[Mesh] OR surrogate* AB OR "Caregivers"[Mesh]) AND ("Clinical Studies as Topic"[Mesh] OR "Clinical Trials as Topic"[Mesh] OR "Controlled Clinical Trials as Topic"[Mesh] OR "Non-Randomized Controlled Trials as Topic"[Mesh] OR "Randomized Controlled Trials as Topic"[Mesh] OR intervention* AB)	1,033
EMBASE	("Advance Care Planning"[Mesh] OR "Decision Making"[Mesh] OR "Advance Directives"[Mesh] OR "Living Wills"[Mesh]) AND ("Family Relations"[Mesh] OR "Family Health"[Mesh] OR "Family"[Mesh] OR surrogate* AB OR "Caregivers"[Mesh]) AND ("Clinical Studies as Topic"[Mesh] OR "Clinical Trials as Topic"[Mesh] OR "Controlled Clinical Trials as Topic"[Mesh] OR "Non-Randomized Controlled Trials as Topic"[Mesh] OR "Randomized Controlled Trials as Topic"[Mesh] OR intervention* AB)	577
PsycINFO (Ovid)	(MM "Advance Directives" OR MM "Decision Making") AND ((MM "Family Relations" OR MM "Family" OR (TI surrogate* OR AB surrogate*) OR MM "Caregivers")) AND ((MM "Clinical Trials" OR MM "Randomized Controlled Trial" OR (TI intervention* OR AB intervention*))	262
Cochrane	(mh "Advance Directives" OR mh "Decision Making") AND (mh "Family Relations" OR mh "Family" OR surrogate*:ti,ab OR mh "Caregivers") AND (mh "Clinical Trials" OR mh "Randomized Controlled Trial" OR intervention*:ti,ab)	284

Study Selection

The literature identified from the database search was uploaded to the Covidence systematic review software (Veritas Health Innovation, Melbourne, Australia). After removing duplicate articles, two independent reviewers (MI and HA) screened the titles and abstracts to assess their suitability for inclusion. Potentially relevant sources were also retrieved, and their citation details were imported into the software. Two independent reviewers (MT and HA) assessed the full texts of the selected citations in detail against the inclusion criteria. The reasons for exclusion were recorded and reported. Disagreements between the reviewers at any stage of the selection process were resolved through discussion. The results of the search and study selection process are reported using the PRISMA 2020 flow diagram.

Risk-of-Bias Assessment

The quality of the selected RCTs was assessed using the Cochrane risk-of-bias tool for randomized trials (RoB) [10]. Briefly, this tool assesses seven items and evaluates internal validity using three levels: “low risk,” “high risk,” and “unclear.” The details are provided in the Appendix. We chose RoB over the revised version (i.e., the Cochrane risk-of-bias tool for randomized trials (RoB2)) owing to its established ease of use [11]. The assessments were performed independently by two reviewers (MT and YI), and any disagreements were resolved by consensus through discussion.

Data Extraction and Synthesis

Data were extracted using a data extraction tool developed by the reviewers. Data, including country, patient, sample size, ACP method, and outcomes (mental health scores), were extracted by two reviewers (MT and YI), and any disagreements were discussed. Each study was synthesized narratively, and key points were summarized and compared. Given the high heterogeneity in ACP interventions, outcomes were summarized by mental health score. Owing to the heterogeneity of the outcome variables and statistical analyses, a meta-analysis was not performed.

Results

Search Results and Study Characteristics

**Figure 1:**
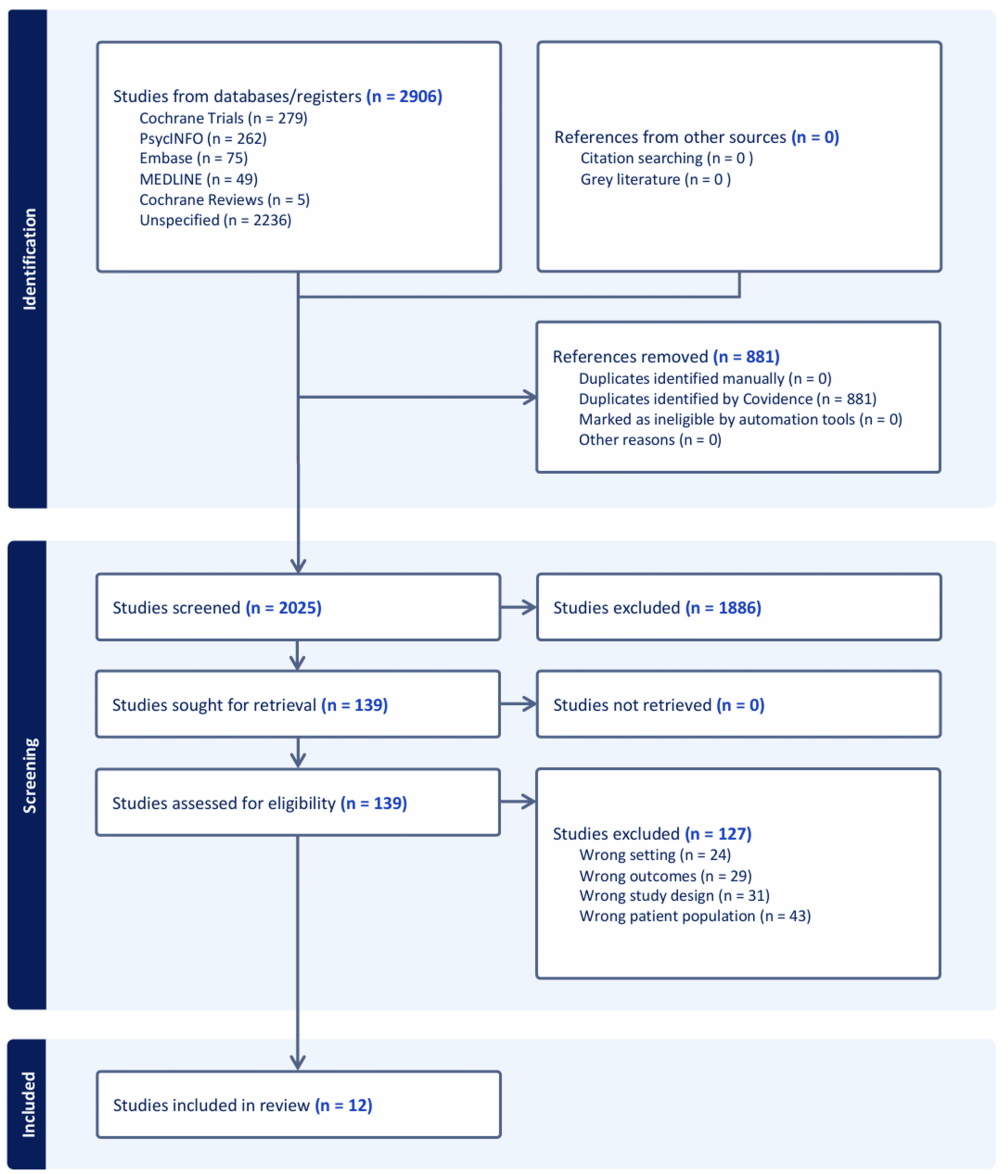
Preferred Reporting Items for Systematic Reviews and Meta-Analyses (PRISMA) flowchart of the study selection process The figure was created by the authors using Covidence (https://www.covidence.org/).

**Figure 2:**
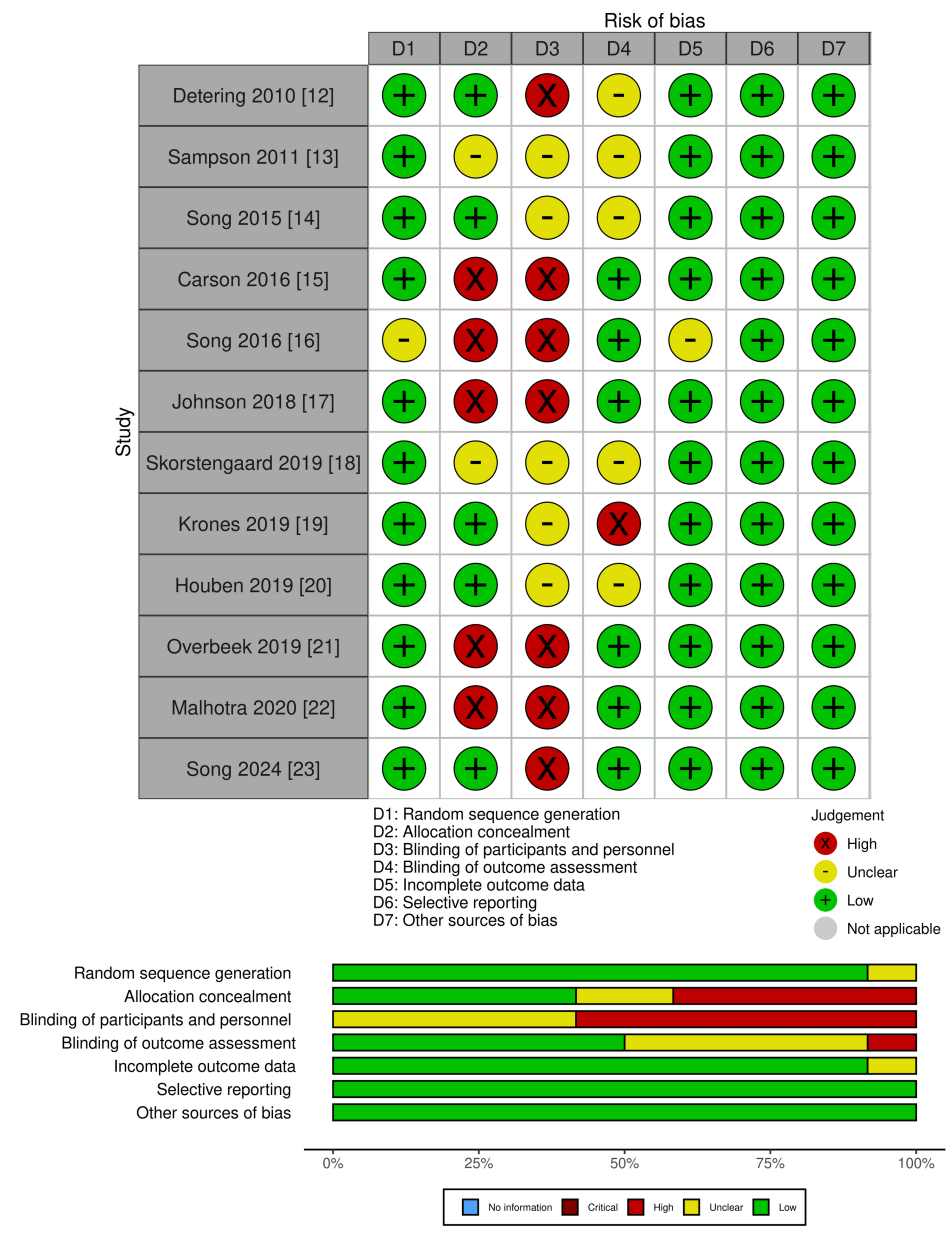
Study quality assessment using the Cochrane risk-of-bias tool for randomized trials The image was created by the authors using robvis (https://www.riskofbias.info/welcome/robvis-visualization-tool).

After excluding duplicates, 2,025 studies were initially evaluated; among these, 1,886 studies were excluded after title and abstract screening. A full-text review of the remaining 139 studies was conducted, and 12 studies published between 2010 and 2024 were selected for final analysis. The study selection flowchart is shown in Figure 1. Of the 12 studies [12-23], four studies were from the USA [14-16,23]; two, the Netherlands [20,21]; two, Australia [12,17]; one, the UK [13]; one, Denmark [18]; one, Switzerland [19]; and one, Singapore [22]. The characteristics of the included studies are summarized in Table 2. The quality assessment of the studies is presented in Figure 2. In RCTs using ACP interventions, many high-risk factors were related to blinding.

**Table 2:** Characteristics of studies included in our systematic review Abbreviations: ACP, advance care planning; COPD, chronic obstructive pulmonary disease; GEE, generalized estimating equation; ICU, intensive care unit; RCT, randomized controlled trial

Author, year	Type of study	Country	Patients	Sample, n	Advance care planning	Mental health-related outcome measures
Detering et al., 2010 [12]	RCT	Australia	Elderly patients	56 intervention = 29, control = 27	Formal ACP by a trained facilitator using the Respecting Patient Choices model	Impact of Events Scale (IES) Hospital Anxiety and Depression Scale (HADS)
Sampson et al., 2011 [13]	RCT	United Kingdom	Patients with severe dementia	33 intervention = 22, control = 10	Palliative care patient assessment, which informed an ACP discussion	Kessler Distress Scale (KD10) Euroqol-5D (EQ-5D)
Song et al., 2015 [14]	RCT	United States	Patients with dialysis	210 intervention = 109, control = 101	ACP intervention that shares the patient’s illness representations to increase trust (SPIRIT)	Hospital Anxiety and Depression Scale (HADS) Post-Traumatic Symptoms Scale-10 (PTSS-10)
Carson et al., 2016 [15]	RCT	United States	Patients with chronic critical illness	256 intervention = 130, control = 126	At least two structured family meetings led by palliative care specialists and provision of an informational brochure	Hospital Anxiety and Depression Scale (HADS) Impact of Event Scale-Revised (IES-R)
Song et al., 2016 [16]	RCT	United States	Patients with dialysis	69 (Whites) intervention = 37, control = 32; 141 (African Americans) intervention = 72, control = 69	ACP intervention that shares the patient’s illness representations to increase trust (SPIRIT)	Hospital Anxiety and Depression Scale (HADS) Post-Traumatic Symptoms Scale-10 (PTSS-10)
Johnson et al., 2018 [17]	RCT	Australia	Patients with incurable cancer	116 intervention = 53, control = 63	ACP intervention was based on the Respecting Patient Choices model, with an offer to provide individualized ranges for typical, best-case, and worst-case scenarios for survival time	Hospital Anxiety and Depression Scale (HADS) Impact of Event Scale-Revised (IES-R) Medical Outcomes Study 12-item Short Form Survey (MOS SF-12)
Skorstengaard et al., 2019 [18]	RCT	Denmark	Patients with lung, heart and cancer diseases	141 intervention = 70, control = 71	ACP discussion to reflect on end-of-life issues, concerns in general, preferred place of care, preferred place of death, preferences for life-prolonging treatment, and cardiopulmonary resuscitation	Symptom Checklist 92-item version (SCL-92)
Krones et al., 2019 [19]	RCT	Switzerland	Patients with severely illness	115 intervention = 57, control = 58	ACP counselling, in which ACP facilitators provide patients and their surrogates with a person-centered discussion about their goals of care	Hospital Anxiety and Depression Scale (HADS)
Houben et al., 2019 [20]	RCT	Netherlands	patients with COPD	165 intervention = 89, control = 76	1.5-hour structured nurse-led ACP-session	Hospital Anxiety and Depression Scale (HADS)
Overbeek et al., 2019 [21]	RCT	Netherlands	Frail older patients	39 intervention = 20, control = 19	Facilitated planning conversations based on the Respecting Choices ACP facilitator training, education materials, and tools	Hospital Anxiety and Depression Scale (HADS)
Malhotra et al., 2020 [22]	RCT	Singapore	Patients with heart failure	282 intervention = 93, control = 189	Trained certified non-clinician facilitators provided ACP based on the Respecting Choices Model	Hospital Anxiety and Depression Scale (HADS) Kansas City Cardiomyopathy Questionnaire (KCCQ)
Song et al., 2024 [23]	ClusterRCT	United States	Patients with dialysis	426 intervention = 231, control = 195	Selected one or two healthcare workers (e.g., nurse practitioner, registered nurse, or social worker) to conduct 45- to 60-minute ACP discussions with dyads in the clinic or remotely.	Hospital Anxiety and Depression Scale (HADS) Post-Traumatic Symptoms Scale-10 (PTSS-10)

Intervention and Evaluation

Patients eligible for ACP included elderly patients; patients on dialysis; and patients with dementia, chronic critical illness, incurable cancer, severe illness, chronic obstructive pulmonary disease (COPD), or heart failure. All interventions involved trained medical professionals to facilitate ACP discussions before the patient died. Mental health was assessed using depression, anxiety, and posttraumatic stress disorder scales.

**Figure 3:**
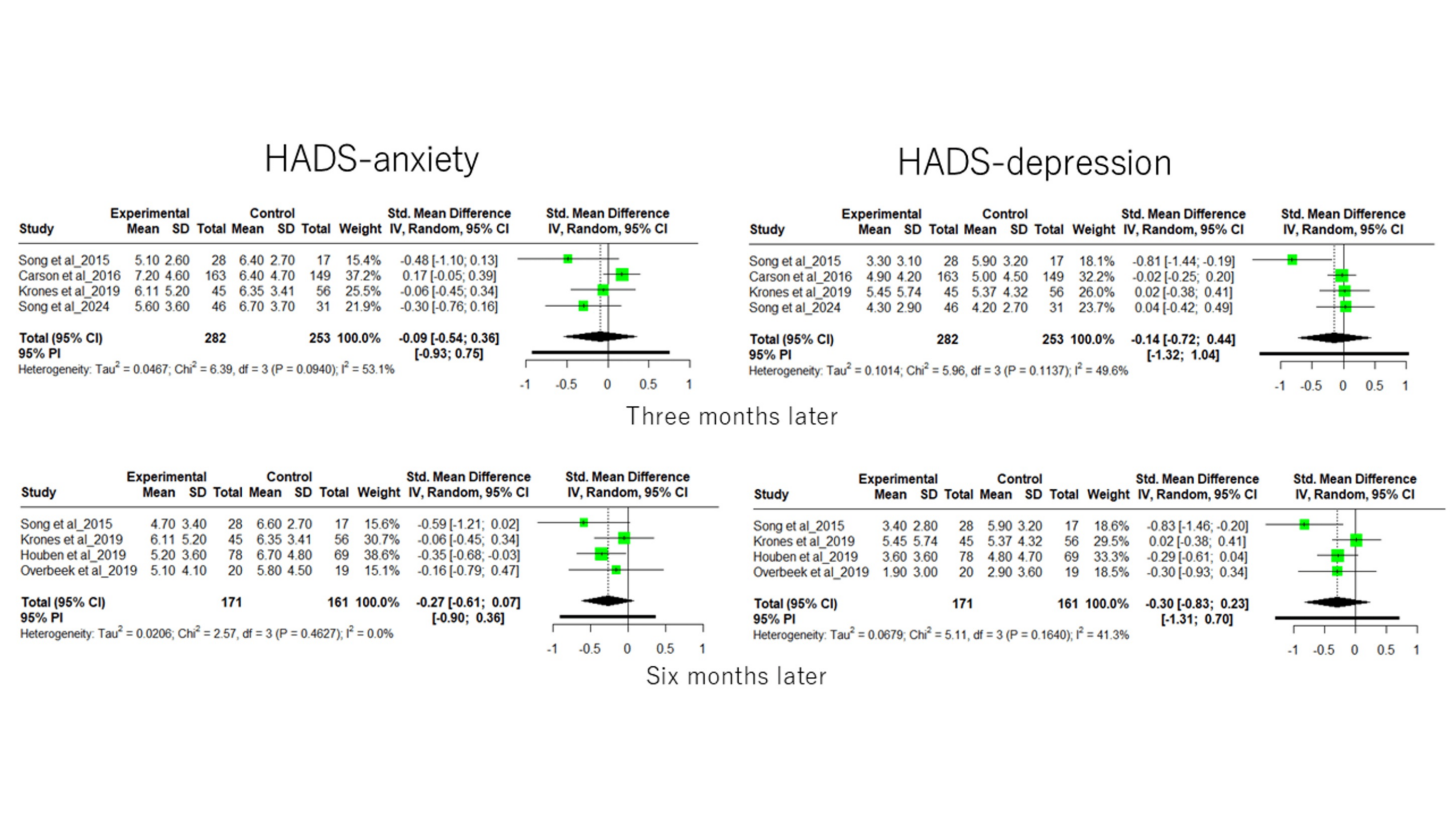
Random-effects model for each outcome The image was created by the authors using R version 4.3.3 (https://www.r-project.org/?2827).

The 12 papers included in this study did not all use common outcomes. In addition, when we checked the data for each outcome, we found that the baseline values and outcomes assessment time differed depending on the paper. Therefore, we attempted to conduct a meta-analysis using a random-effects model. To evaluate overall heterogeneity, we calculated I2, Q test, and predicted interval for each outcome and examined heterogeneity. As a result, heterogeneity was observed for all outcomes. The results are shown in Figure 3. Taking into account the baseline and outcomes, we determined that it was not appropriate to conduct a subgroup analysis. Therefore, in order to prevent misleading results, this study was limited to a systematic review.

Effectiveness of an ACP Intervention

The items for which significant differences were reported varied among the studies, but several studies reported effectiveness in treating depression among bereaved families [12,14,16]. Furthermore, when baseline-adjusted results were included, ACP interventions reduced post-bereavement anxiety [12,14,20,23] and stress [12,14] experienced by bereaved families. Meanwhile, some studies reported no significant improvements in the mental health scores of bereaved families in response to ACP interventions [13,18,19,21,22]. Furthermore, one study showed that ACP intervention resulted in increased stress for bereaved families [15]. The effectiveness of an ACP intervention is shown in Table 3.

**Table 3:** Effectiveness of ACP interventions on mental health-related outcomes in each study Abbreviations: QOL, quality of life *After adjusting for time and the baseline value, there was a significant intervention effect on reducing depressive symptoms for African Americans (β = -3.49 [95% CI, -5.82 to -1.17]; P = 0.003).

Author, year	Anxiety	Depression	Stress	QOL
Detering et al., 2010 [12]	Decreased	Decreased	Decreased	―
Sampson et al., 2011 [13]	No effect	No effect	―	No effect
Song et al., 2015 [14]	Decreased	Decreased	Decreased	―
Carson et al., 2016 [15]	No effect	No effect	Increased	―
Song et al., 2016 [16]	No effect	Decreased *	No effect	―
Johnson et al., 2018 [17]	No effect	No effect	No effect	Decreased
Skorstengaard et al., 2019 [18]	No effect	No effect	―	―
Krones et al., 2019 [19]	No effect	No effect	―	―
Houben et al., 2019 [20]	Decreased	No effect	―	―
Overbeek et al., 2019 [21]	No effect	No effect	―	―
Malhotra et al., 2020 [22]	No effect	No effect	―	No effect
Song et al., 2024 [23]	Decreased	No effect	No effect	―

Discussion

This study clarifies the impact of ACP interventions on the mental health of bereaved families. The results show that ACP may be effective in improving the mental health of bereaved families. Previous systematic reviews have focused on increased discussions [24], but to our best knowledge, this is the first review to focus on the mental health of bereaved families as an outcome.

Many studies have reported that ACP interventions had a positive effect on bereaved families, suggesting that they are particularly effective in reducing depression and anxiety after bereavement. Interestingly, similar effects have been observed across studies. Research on ACP, not limited to RCTs, is being conducted worldwide [25-28]. The ACP intervention efforts in the included reports in this study were not limited to a specific country or region. Moreover, the intervention targets were diverse and included elderly patients; patients on dialysis; and patients with dementia, chronic critical illness, incurable cancer, severe illness, COPD, or heart failure. ACP is a process of discussing a patient’s future care [28], and it targets a diverse population. Accordingly, this process is likely to take various forms depending not only on illness and age but also on culture and views on life and death. However, ACP interventions can positively affect the mental health of the bereaved family of any patient.

Approximately 50% of bereaved families experience prolonged grief disorder [29], and ACP interventions may address this problem. However, some studies have also reported no significant improvements in the mental health scores of bereaved families following ACP intervention [17,19,21,22]. No clear regional characteristics (Australia, Switzerland, Netherlands, and Singapore) or patient diseases (cancer, severe illness, frailty, heart failure) were observed in these studies. The effectiveness of ACP intervention reportedly decreases over time [30], and the timing of the intervention also needs to be verified. Future research should verify the type of intervention that leads to improvements in mental health.

Limitations

This study had limitations. First, a meta-analysis was not applicable because of the heterogeneity of the outcome variables and the statistical analysis. Therefore, this study was limited to a narrative synthesis of evidence. It is expected that meta-analysis will become possible in the future as more studies using common outcomes are conducted. Second, this review was limited to studies published in English, which may have resulted in the exclusion of relevant research published in other languages. As a consequence, findings from non-English-speaking regions may not be fully represented, potentially limiting the generalizability of our results to diverse global contexts. In the future, it is hoped that language constraints will be alleviated by conducting systematic reviews in collaboration with researchers from various countries.

## Conclusions

Depending on the targets and intervention methods, pre-death discussions are expected to improve the mental health of bereaved families. ACP can also support decision-making during the bereavement process, which is mentally stressful. ACP implementation may contribute to solving the pressing mental health issues of bereaved families. Thus, ACP should be implemented to help bereaved families. On the other hand, there is a lack of evidence supporting the effectiveness of ACP for bereaved families. It became clear that research using common outcomes and setting a baseline assessment time will become more important issues. Furthermore, if language limitations can be improved, it is expected that regional characteristics and new evidence will become clearer.
